# Fungal and parasitic contamination of indoor public swimming pools in Arak, Iran

**DOI:** 10.1186/s42506-020-0036-3

**Published:** 2020-03-17

**Authors:** Hossein Sarmadian, Yasamin Hazbavi, Mojtaba Didehdar, Mohammad Javad Ghannadzadeh, Reza Hajihossein, Mahmood Khosravi, Reza Ghasemikhah

**Affiliations:** 1 Department of Infectious Disease, Arak University of Medical Sciences, Arak, Iran; 2 Department of Parasitology and Mycology, Arak University of Medical Sciences, Arak, Iran; 3 Department of Environmental Health Engineering, Arak University of Medical Sciences, Arak, Iran; 4 School of Paramedical Sciences, Arak University of Medical Sciences, Arak, Iran

**Keywords:** *Acanthamoeba*, Arak, Fungi, Physicochemical parameters, Public swimming pools

## Abstract

**Background:**

Swimming is a popular exercise for different types of people at different ages. Public swimming pools are places where fungal infections can be easily transferred. The purpose of this study is to evaluate the quality of mycological, parasitological, and physicochemical parameters of swimming pools of Arak city.

**Methods:**

This cross-sectional study was done for 12 months from April 2013 to March 2014 in six indoor active swimming pools of Arak city (A, B, C, D, E, and F). Samples were collected in four seasons, two times/season; each time, two samples were obtained from six specified locations (shallow level pool, deep level pool, dressing rooms, showers, margin of pool walls, and foot-washing sink) from each pool with a total of 576 samples. Physicochemical parameters including water temperature, pH, turbidity, and the residual chlorine were measured on-site. In order to isolate and detect the fungal agents, special filters and culture Sabouraud’s dextrose agar, chloramphenicol, and mycosel agar media were applied. Furthermore, non-nutrient agar medium enriched with *Escherichia coli* was used to detect and to separate the eggs of the worms, cysts, and parasitic protozoa from centrifuges of samples. In order to investigate their sediment, optical microscope and culture media were used.

**Results:**

We found that 456 (79.1%) samples were positive regarding the fungal elements, and 516 fungal species were isolated. The most common isolates were saprophytic species (8.74%), yeast species (25%), and dermatophyte species (2.5%). The most contaminated surfaces were foot-washing sinks and showers. In this study, *Acanthamoeba* parasites were isolated from one pool only.

All the investigated physicochemical parameters of pool water except for the temperature were found to be in the standard range.

**Conclusions:**

Existence of saprophytic fungi and yeast in pools’ water is plausible to be considered as an indicator of water resistance to the detergent agents. This high degree of contamination is due to the huge number of visitors, the complexity of construction, the choice of materials, and the long opening hours. Isolation of dermatophytes and *Acanthamoeba* parasite from the pools’ area and foot-washing sink reveals the important role of the public swimming pools in disease transmission.

## Introduction

Swimming is an excellent way to get the physical activity and health benefits needed for having a healthy life at all ages. However, the cleanliness of the water and its management should be guaranteed [1]. Swimming pools are usually accompanied with potentially hazardous health issues. Indeed, given that they are being used by many people within a restricted time period, they are a suitable place for transferring infections and skin diseases. Pool-borne communicable diseases include bacterial infections such as eye infections, external otitis, parasitic diseases such as giardiasis and cryptosporidiosis, and fungal diseases such as dermatophytosis and opportunistic mycosis [2–6]. Therefore, swimming pool pollution has become a growing concern.

These fungi are causing a spectrum of infections including dermatophytosis [7]. If someone with this infection goes to a swimming pool, he has a direct role in transferring diseases and infections to others [8]. Humidity, heat, and excessive heating can also be responsible for the transfer of fungal skin diseases. Hence, taking a shower before and after swimming and using a personal swimming cap, towel, and slippers are necessary, and those should not be shared with others. Those who are infected with dermatophytosis should not go swimming given that direct contact is another way of transferring diseases.

Evidence-based data show that swimming pools are suitable places for the growth of pathogenic fungi if they are not controlled or examined [4, 9]. Surfaces of pool bottoms and edges, locker rooms, and showers are also suitable for the growth of dermatophyte fungi; other at-risk places include hot tubs given that saprophytic fungi can easily grow due to the humidity of the environment [10–12]. Opportunistic fungi have pathogenic potential for immunodeficient and immunocompromised subjects [10].

*Acanthamoeba* is a free-living opportunistic amoeba that has a global spread and is widely distributed in water, soil, air, sewage, and other environments [13]. *Acanthamoeba* species are observed in two forms of trophozoite and cysts. *Acanthamoeba* cyst is resistant to the chlorination and disinfection of water systems. The habitat of *Acanthamoeba* is water and especially pool water [13, 14]. This amoeba can cause *Acanthamoeba* keratitis and central nervous system infection. There have been reports of people with keratitis who had swimming experiences in the pools [15].

The assessment of physicochemical parameters of swimming pool waters such as pH, temperature, and residual chlorine has crucial role in water quality and pathogenic microorganism control [16]. Standard ranges of physicochemical parameters for swimming pool waters are as follows: pH between 7.2 and 8, temperature between 29 and 25 °C, and turbidity less than 0.5 NTU (Nephelometric Turbidity Unit) and for residual chlorine 1–3 ppm (parts-per-million).

In alkaline pH, less amount of the residual chlorine changes into hydrochloric acid and the disinfection effects became weaker [1, 17]. Using materials other than chlorine as a disinfectant can lead to the transfer of different types of diseases in public places. Acidic pH of swimming pool water can lead to eye irritation and dry hair and skin and reduces the amount of residual chlorine [1].

Considering the importance of studying the fungal and parasitic contamination of swimming pools and the fact that no such study has been carried out in Arak city so far, therefore, the present study was performed to evaluate the fungal and *Acanthamoeba* contamination and physicochemical quality parameters in swimming pools in Arak city in the center of Iran.

## Methods

### Study design, study setting, and sampling technique

This cross-sectional study was done for 12 months from April 2013 to March 2014 in six indoor active swimming pools of Arak city (A, B, C, D, E, and F). Samples were collected in four seasons, two times/season; each time, two samples were obtained from six specified locations (shallow level pool, deep level pool, dressing rooms, showers, margin of pool walls, and foot-washing sink) from each pool with a total of 576 samples [18]. Sample from females’ swimming pools were collected by a female sampler and from males’ swimming pools by a male sampler.

Water samples were collected by using sterilized bottles from shallow and deep levels for physical and chemical parameters and mycological and parasitological analysis. In addition, four samples from dressing rooms, showers, margin of pool walls, and foot-washing sink were taken by using a piece of sterile carpet (size of 5 × 5 cm^2^). The carpets were placed in sterile nylons. All samples were immediately transferred to the laboratory.

### Mycological investigations

Samples of 100 ml of collected water were passed through 45 μm Millipore filters (Merck, Germany). Filters were put on Sabouraud’s dextrose agar (SDA) with chloramphenicol and mycosel agar (Merck, Germany). Also, the carpets were shaken and put on Sabouraud’s dextrose agar with chloramphenicol and mycosel agar. The media were incubated at 28 °C for 3 weeks and investigated daily. Filamentous fungal isolates were identified by slide culture method [19]. Yeast fungal isolates were subcultured on CHROMagar™ Candida medium (CHROMagar, France) and incubated at 35 °C for 48 h. Discrimination of *Candida* species and other yeast species was done according to different colors produced by *Candida* colonies in the chromogenic medium according to the manufacturer’s protocol [20].

### Parasitic investigations

For isolation of *Acanthamoeba* parasite, 100 ml of sampled waters was passed through 45 μm size Millipore filters (Merck, Germany). Filters were put on a non-nutrient agar medium enriched with *Escherichia coli* [21]. Then, the lid of the medium plate was blocked with paraffin and incubated in a 28 °C for 10 days. Afterwards, the plate was examined under a microscope with magnification of four times and ten times for *Acanthamoeba* colony.

For isolation of other parasites, 200 ml of pool water was centrifuged at 4000 rpm for 10 min. Direct smear was prepared from precipitate and examined under a microscope with magnification of 10 times and 40 times for egg, cyst, and trophozoite of parasites.

### Physicochemical analysis

Physicochemical parameters including water temperature, pH, turbidity, and chlorine residual were measured on-site. Residual chlorine was determined with DPD (Plain test, USA) instrument, and pH was measured by general method. Water turbidity and temperature were determined using turbidimeters (HACH, USA) and a thermometer (HACH, USA) respectively.

### Statistical analysis

SPSS version 13 was used for data analysis. Chi-squared test and one-way ANOVA were used. *p* < 0.05 was considered the level of significance.

## Results

### Percentage of fungal contamination and isolated fungi

In this study, 456 (79.1%) samples out of 576 collected samples were positive for fungal elements, and 516 fungal species were isolated. Our results indicate that the most common fungal isolates include 412 saprophytic fungi (79.8%), 103 yeast species (20%), and 1 dermatophyte species (0.2%).

Table 1 shows that the most fungal contamination was observed in the swimming pools A and B and the least one in the pool E. The most common isolated fungal species were as follows: 103 *Candida* species (20%), 86 *Penicillium* species (16.7%), and 84 *Aspergillus* species (16.3%). One case of the dermatophyte species which was isolated from foot-washing sink of the swimming pool C was *Trichophyton rubrum* (Tables 1 and 2). The most common species of the saprophytic fungi and yeast species that were isolated from the water of swimming pools include *Aspergillus* and *Candida* species (Table 2).

**Table 1:** Frequency of fungal species isolated regarding the type of swimming pool

Fungi species	Type of pool							*p* value*
	A	B	C	D	E	F	Total	
	No. (%)	No. (%)	No. (%)	No. (%)	No. (%)	No. (%)	No. (%)	
*Candida* spp.	25 (26)	19 (20)	13 (17)	17 (20)	12 (17)	17 (19)	103 (20)	> 0.05
*Candida albicans*	19 (20)	14 (15)	10 (13)	14 (16)	10 (14)	13 (14)	80 (15.5)	> 0.05
*Candida parapsilosis*	5 (5)	4 (4)	2 (3)	3 (3)	2 (3)	4 (4)	20 (4)	> 0.05
*Candida krusei*	1 (1)	1 (1)	1 (1)	0	0	–	3 (0.5)	> 0.05
*Penicillium* spp.	16 (17)	18 (19)	11 (15)	16 (19)	12 (17)	13 (14)	86 (16.7)	> 0.05
*Aspergillus* spp.	15 (16)	17 (18)	10 (13)	14 (16)	14 (19)	14 (15)	84 (16.3)	> 0.05
*Alternaria* spp.	9 (9)	14 (15)	13 (17)	8 (9)	10 (14)	14 (15)	68 (13.2)	> 0.05
*Fusarium* spp.	10 (10)	10 (10)	5 (7)	13 (15)	9 (13)	15 (16)	62 (12)	> 0.05
*Cladosporium* spp.	4 (4)	5 (5)	9 (12)	5 (6)	6 (8)	9 (10)	38 (7.3)	> 0.05
*Rhodotorula* spp.	5 (5)	5 (5)	5 (7)	3 (3)	4 (6)	4 (4)	26 (5)	> 0.05
*Rhizopus* spp.	7 (7)	3 (3)	4 (5)	3 (3)	2 (3)	2 (2)	21 (4)	> 0.05
*Mucor* spp.	3 (3)	3 (3)	3 (4)	6 (7)	1 (1)	2 (2)	18 (3.5)	> 0.05
*Drechslera* spp.	1 (1)	1 (1)	1 (1)	–	–	1 (1)	4 (0.8)	–
*Curvularia* spp.	1 (1)	1 (1)	–	–	1 (1)	–	3 (0.6)	–
*Trichophyton rubrum*	–	–	1 (1)	–	–	–	1 (0.2)	–
*Scopulariopsis* spp.	–	–	–	1 (1)	–	–	1 (0.2)	–
*Acremonium* spp.	–	–	–	–	1 (1)	–	1 (0.2)	–
Total no. (%)	96 (18.6)	96 (18.6)	75 (14.5)	86 (16.7)	72 (14)	91 (17.6)	516 (100)	> 0.05

*ANOVA

The mean difference is significant at the 0.05 level

**Table 2:** Distribution and frequency of isolated fungi species according to place of sampling

Fungi species	Place of sampling						*p* value*
	Pool water	Foot-washing sink	Margin of pool walls	Shower	Dressing room	Total (%)	
	No. (%)	No. (%)	No. (%)	No. (%)	No. (%)	No. (%)	
*Candida* spp.	5 (23)	25 (19)	20 (17)	37 (28)	16 (14)	103 (20)	> 0.05
*Candida albicans*	5 (23)	20 (15)	15 (13)	30 (23)	10 (9)	80 (15.5)	> 0.05
*Candida parapsilosis*	0 (0)	5 (4)	4 (3)	6 (5)	5 (5)	20 (4)	> 0.05
*Candida krusei*	0 (0)	0 (0)	1 (1)	1 (1)	1 (1)	3 (0.5)	> 0.05
*Penicillium* spp*.*	0 (0)	20 (15)	20 (17)	17 (13)	29 (26)	86 (16.7)	> 0.05
*Aspergillus* spp.	6 (27)	17 (13)	16 (13)	18 (14)	27 (24)	84 (16.3)	> 0.05
*Alternaria* spp.	1 (5)	18 (14)	14 (12)	21 (16)	14 (13)	68 (13.2)	> 0.05
*Fusarium* spp.	0 (0)	20 (15)	18 (15)	13 (10)	11 (10)	62 (12)	> 0.05
*Cladosporium* spp.	3 (14)	10 (8)	15 (13)	2 (2)	8 (7)	38 (7.3)	> 0.05
*Rodotorula* spp.	3 (14)	10 (8)	4 (3)	8 (6)	1 (1)	26 (5)	> 0.05
*Rhizopus* spp.	2 (9)	5 (4)	3 (3)	9 (7)	2 (2)	21 (4)	> 0.05
*Mucor* spp.	1 (5)	6 (5)	7 (6)	3 (2)	1 (1)	18 (3.5)	> 0.05
*Drechslera* spp.	0 (0)	1 (1)	0 (0)	2 (2)	1 (1)	4 (0.8)	> 0.05
*Curvularia* spp.	1 (5)	0 (0)	1 (1)	1 (1)	–	3 (0.6)	–
*Trichophyton rubrum*	0 (0)	0 (0)	0 (0)	0 (0)	–	1 (0.2)	–
*Scopulariopsis* spp.	0 (0)	0 (0)	1 (1)	0 (0)	–	1 (0.2)	–
*Acremonium* spp.	0 (0)	0 (0)	0 (0)	0 (0)	1 (1)	1 (0.2)	–
Total (%)	22 (4.3)	133 (25.5)	119 (23)	131 (25.4)	111 (21.5)	516 (100)	> 0.05

*ANOVA

The mean difference is significant at the 0.05 level

According to our findings, the most contaminated surfaces are foot-washing sinks and showers. The most frequently isolated species from these places were *Candida*, *Penicillium*, and *Aspergillus* species (Table 2).

As it is demonstrated in (Table 3), the fungal contamination of the swimming pools was higher in the summer season than any other times. The most isolated fungal species in this season were *Candida* species, *Penicillium* species, *Aspergillus* species, and *Fusarium* species. Among the isolated *Candida* species, the most common species were *C*. *albicans*, *C*. *parapsilosis*, and *C*. *krusei*, respectively (Tables 1, 2, and 3).

**Table 3:** Distribution of isolated fungi species according to season

Fungal species	Season					*p* value*
	Spring	Summer	Fall	Winter	Total (%)	
	No. (%)	No. (%)	No. (%)	No. (%)	No. (%)	
*Candida* spp.	17 (15)	45 (26)	22 (18)	19 (18)	103 (20)	> 0.05
*Candida albicans*	15 (13)	37 (22)	15 (12)	13 (12)	80 (15.5)	> 0.05
*Candida parapsilosis*	1 (1)	8 (5)	5 (4)	6 (6)	20 (4)	> 0.05
*Candida krusei*	1 (1)	0 (0)	2 (2)	0 (0)	3 (0.5)	> 0.05
*Penicillium* spp.	18 (16)	25 (15)	23 (19)	20 (19)	86 (17)	> 0.05
*Aspergillus* spp.	21 (18)	25 (15)	22 (18)	16 (15)	84 (16)	> 0.05
*Alternaria* spp.	17 (15)	16 (9)	20 (16)	15 (14)	68 (13)	> 0.05
*Fusarium* spp.	15 (13)	22 (13)	10 (8)	15 (14)	62 (12)	> 0.05
*Cladosporium* spp.	6 (5)	12 (7)	15 (12)	5 (5)	38 (7)	> 0.05
*Rodotorula* spp.	5 (4)	9 (5)	7 (6)	5 (5)	26 (5)	> 0.05
*Rhizopus* spp*.*	12 (10)	4 (2)	0 (0)	5 (5)	21 (4)	> 0.05
*Mucor* spp.	2 (2)	8 (5)	4 (3)	4 (4)	18 (3.5)	> 0.05
*Drechslera* spp.	0 (0)	2 (1)	1 (1)	1 (1)	4 (0.8)	–
*Curvularia* spp.	1 (1)	1 (1)	0 (0)	1 (1)	3 (0.6)	–
*Trichophyton rubrum*	0 (0)	1 (0)	0 (0)	0 (0)	1 (0.2)	–
*Scopulariopsis* spp.	1 (1)	0 (0)	0 (0)	0 (0)	1 (0.2)	–
*Acremonium* spp.	0 (0)	–	0 (0)	1 (1)	1 (0.2)	–
Total (%)	115 (22.3)	170 (33)	124 (24)	107 (20.7)	516 (100)	> 0.05

*ANOVA

The mean difference is significant at the 0.05 level

### Percentage of parasitic contamination

*Acanthamoeba* parasite was detected in only one pool from the six studied pools; it was mainly detected in foot-washing sinks, surface water, and deep water.

### Sanitary parameters

Physical and chemical parameters were in standard range except for the temperature parameter (Table 4); among the studied swimming pools, the highest and the lowest pH levels were in pools A and C (Table 4). Residual chlorine in the winter and summer was high, and swimming pools had higher levels of turbidity in the summer than in other seasons (Table 4).

**Table 4:** Mean of physicochemical parameters of swimming pool water according to type of swimming pool and season

Physicochemical parameter	Type of swimming pool						*p* value*	Season				*p* value*
	A	B	C	D	E	F		Spring	Summer	Fall	Winter	
pH	7.529 ± 0.2	7.386 ± 0.3	7.157 ± 0.1	7.386 ± 0.2	7.357 ± 0.4	7.343 ± 0.1	> 0.05	7.3 ± 0.2	7.5 ± 0.2	7.3 ± 0.2	7.5 ± 0.2	> 0.05
Residual chlorine	1.9 ± 0.7	2.4 ± 0.5	1.6 ± 0.6	1.8 ± 0.3	1.6 ± 0.4	2.1 ± 0.6	< 0.05	1.8 ± 0.3	1.8 ± 0.8	1.8 ± 0.3	1.8 ± 0.8	> 0.05
Turbidity	729.0 ± 101.9	802.8 ± 188.2	867.1 ± 29.3	484.9 ± 261.1	833.1 ± 97.7	723.0 ± 128.2	< 0.05	713.7 ± 237	721.2 ± 246	750.8 ± 208	797.8 ± 120	> 0.05
Temperature	29.3 ± 0.5	29.5 ± 0.4	29.9 ± 0.4	29.1 ± 0.4	29.9 ± 0.4	29.9 ± 0.4	< 0.05	29.7 ± 0.5	29.7 ± 0.5	29.5 ± 0.5	29.5 ± 0.5	> 0.05

*ANOVA

The mean difference is significant at the 0.05 level

## Discussion

Swimming pools are suitable places for transferring the pathogenic and potentially pathogenic microorganisms to human [22]. Factors such as swimmers’ skin infections along with the lack of pH control and inadequate disinfection of swimming pools have an important role in transferring infectious diseases [16].

In the present study, physiochemical parameters of swimming pools, water fungal contamination, and pools’ surroundings in Arak were investigated. Results showed that fungi species were found in 79.1% of swimming pools’ water or surface samples. These species were saprophytic fungi (74.8%), yeast species (25%), and dermatophytes species (0.2%).

Several studies in different parts of Iran and the world were done to investigate pool contamination [4, 8, 9, 11, 16, 17, 23–27]. Similarly to other studies in Iran, we found that saprophytic filamentous fungi were the most prevalent [11, 17, 23, 24] and *Candida* species were the most common fungal isolate. Similar to Rasti in Kashan and Jahanbakhsh, in Urmia, we found that fungal contamination was more common during the summer season, and the least problematic in the spring due to the fact that pools have more swimmers in summer and the heat and humidity are higher in this season [17].

It is in contrast to the study by Rafiei which found that 54.47% of the total collected samples were positive regarding the fungi elements. However, the corresponding rate of fungal contamination of all collected samples in the present study was more than what had been found in that study. Furthermore, the most common isolates are filamentous fungi in that study which is similar to our results [9].

Compared with the other studies in Iran [9, 17, 20], the lowest rate of dermatophyte species was isolated in the current study which can be due to the hygiene of the swimmers of our considered pools. In the present study, only one case of *Trichophyton rubrum* was isolated from foot-washing sink. In addition, in contrast to some previous studies in Iran [9, 11, 24] and elsewhere [4, 8, 26], no case of dermatophyte species was isolated from lockers, showers, pool edges, and water, which could be explained by our residual chlorine and isolation method.

In the study of Jankowski in Poland that was done on swimming pools, in contrast to the study, *T*. *mentagrophytes* was the most prevalent dermatophyte isolated, while only one species of *Microsporum canis* was isolated and no case of *T*. *rubrum* was isolated, which was detected in 86% of all adult dermatophytic infections. Also similar to our study, in the study of Jankowski, the most isolated species were *Candida* spp. [28].

In the study of Brandi in Rome city of Italy, in contrast to other studies, *Candida* spp. has been never detected, while similar to the present study, one species *T*. *rubrum* was isolated from swimming pools’ water and surfaces [26].

Nowadays, diseases caused by free-living amoeba including encephalitis and keratitis are increasing [29]. In our country, various studies have been done on free-living amoeba in environmental resources including pool water. In the present study, similar to other studies in Iran and the world and in contrast to the study of Armand in Shiraz, one case of *Acanthamoeba* contamination out of six pools (16.6%) was isolated. In the present study, *Acanthamoeba* was isolated in the warm season (summer) of the year where many people are using the pools, which could be the cause of high rates of contamination [30–33].

In a study by Mafi et al. on pool water and pools of amusement parks in Tehran, they reported that the contamination rate with *Acanthamoeba* was 24%, which is different from the results of our study, due to the low level of contamination in this study that could be due to sampling only from the pool water, while in Mafi et al.’s study, in addition to pool water, samples were collected from the park ponds, due to being in open space and also entering of the dust to these waters which has increased the amount of *Acanthamoeba* contamination [34].

In a study conducted by Solgi et al. on the hot springs of Ardabil province, 20% of the hot springs of this province were contaminated with *Acanthamoeba* parasite. The reason for the difference in the results of our study and that study can be attributed to the difference in water temperature where the water temperature of the hot springs is higher than that of the pool water [35]. The results of the present study indicate that *Acanthamoeba* free-living amoeba is significantly prevalent in Arak city pools and there is a potential for infection to people who are prone to free-living amoeba.

Our finding showed that the pools’ water pH was between 7.15 and 7.52, which is in accordance with the recommended reference pH for swimming pool water (7.2–8.0). The pool water pH in this study in comparison to other studies in Iran was in a better condition. In addition, unlike other studies in Iran [17, 36, 37], the residual chlorine of all swimming pools was in the standard range (1–3 mg/L). The water turbidity of the swimming pools in this study was less than 0.5 NTU, which was in the standard condition. In contrast to other physicochemical parameters in the present study and similar to study of Rasti in Kashan [16], the water temperature of swimming pools was more than the standard range (25–29 °C). It is known that high water temperature provides conditions for the growth of pathogenic microorganisms [35, 38].

## Conclusion and recommendations

The present study revealed a higher degree of saprophytic fungal contamination of swimming pools with potentially pathogenic *Acanthamoeba*. Therefore, it is essential to pay closer attention to the hygiene and safety of these swimming pools’ water sources.

Based on our findings, the dermatophytic contamination of investigated swimming pools was low and physiochemical parameters except from the water temperature were in the standard range. However, saprophytic fungal contamination of the swimming pools was significant which could be due to the lack of the showering before entering the pool and also to the high temperature of the pool water. Therefore, it is essential to inform the swimmers to improve their knowledge and hygienic behavior.

*Acanthamoeba* was detected in 16.6% of the swimming pools, confirming the widespread distribution of these free-living amoeba in the environment and the risks associated with the contamination of contact lens wearers and immunocompromised patients.

Concerning the water contamination of the pools with *Acanthamoeba* parasite and in order to prevent the occurrence of the corresponding infections, necessary warnings should be made by the health authorities about the precise disinfection of the pools and appropriate strategies for dealing with free-living amoeba should be applied. Moreover, the continuous monitoring of physicochemical parameters especially the residual chlorine and the water temperature is advised to maintain the quality of the pool water.

## Data Availability

In this study, all data and materials are included. If more information is needed, please contact the author for data requests.
